# Primitive reflexes as behavioral biomarkers of cognitive aging: associations with physical activity and resilience—a pilot study

**DOI:** 10.3389/fnagi.2025.1687512

**Published:** 2025-11-26

**Authors:** Anna Horváth-Pápai, Eliza Eszter Tóth, István Barthalos, Zoltán Alföldi, Johanna Takács, Ferenc Ihász

**Affiliations:** 1 Doctoral School of Health Sciences, Faculty of Health Sciences, University of Pécs, Pécs, Hungary; 2 Faculty of Health and Sport Sciences, Széchenyi István University, Győr, Hungary; 3 Doctoral School of Psychology, Faculty of Education and Psychology, Eötvös Loránd University, Budapest, Hungary; 4 Department of Social Sciences, Faculty of Health Sciences, Semmelweis University, Budapest, Hungary

**Keywords:** primitive reflexes, cognitive aging, physical activity, behavioral biomarkers, executive function

## Abstract

**Introduction:**

Primitive reflexes (PRs) are brainstem-mediated automatic responses that typically disappear in early life, but may reappear in older age, which may be associated with neurodegenerative processes. But the presence of PRs in cognitively healthy adults has not yet been sufficiently explored. The relationship between PRs and cognitive functioning (COG) may be influenced by modifiable factors such as physical activity (PA) and psychological resilience. This cross-sectional observational pilot study aimed to investigate the mediating and moderating role of physical activity and resilience in the association between primitive reflexes and cognitive functioning in older adults.

**Methods:**

A total of 30 older adults (mean age 73.4 ± 6.9 years; 80% female) living in residential care facilities were assessed. PRs were evaluated using standardized neurological protocols, COG was measured with the Mini-Mental State Examination, PA with the Global Physical Activity Questionnaire, and resilience with the Connor–Davidson Resilience Scale. Moderation and mediation models were tested using Hayes’ PROCESS macro, controlling for age and BMI.

**Results:**

A higher number of primitive reflexes was strongly associated with lower cognitive functioning [COG (*r* = −0.904, *p* < 0.001)]. Physical activity showed a significant mediating effect in this association, indicating that more active older adults exhibited better cognitive performance despite the presence of primitive reflexes. Resilience, although correlated with both cognition and physical activity, did not show a mediating or moderating effect.

**Discussion:**

These findings highlight primitive reflexes as potential behavioral biomarkers of cognitive aging, and underscore the importance of physical activity as a protective factor that may buffer against neurocognitive decline.

## 1 Introduction

Primitive reflexes are complex, automatic movement patterns mediated by the brain stem. They develop in the third trimester of pregnancy and probably disappear in the first three quarters of life (Zafeiriou, 2004). They may reappear as symptoms of certain neurodegenerative diseases. The cessation of primitive reflex activity is associated with the maturation of neural networks, particularly those related to frontal brain structures (Isakov et al., 1984; Jolles et al., 1993). Many researchers refer to the reappearance of reflex activity as “retrogression” (de Ajuriaguerra et al., 1963; Reisberg et al., 1999). It remains an open question whether recurrent primitive reflexes are physiological companions of aging. The integrity of prefrontal networks is crucial for complex actions such as behavioral planning and the performance of tasks that rely on working memory and executive control (Miller, 2000). Since the above-mentioned brain structures are particularly sensitive to aging (Miller and Cohen, 2001), it can be assumed that age-related cognitive decline and the appearance of primitive reflexes are consequences of the increased frequency of the same oxygen-deficient areas (Schott and Rossor, 2003). Age-related cognitive decline has been linked to structural and functional alterations in the frontal and hippocampal regions, which are particularly sensitive to reductions in cerebral blood flow and oxygen metabolism. With advancing age, cerebral perfusion decreases, especially in the prefrontal cortex and subcortical white matter (Farkas and Luiten, 2001). Chronic, low-grade cerebral hypoperfusion can lead to localized oxygen deficiency and neuronal energy imbalance, impairing synaptic transmission and executive functioning (Iadecola, 2013). Because primitive reflexes are normally inhibited by intact cortico-subcortical networks—particularly those involving the frontal lobes—hypoxic or ischemic damage to these regions may reduce cortical inhibition, allowing brainstem-mediated reflexes to re-emerge. Thus, both age-related cognitive decline and the reappearance of primitive reflexes may stem from shared hypoxia-related mechanisms affecting similar cortical and subcortical areas. Based on these findings, the question arises whether the presence of primitive reflexes in healthy older adults can be associated with impairments in certain cognitive functions, such as memory and information processing speed. The vast majority of research on this topic has been cross-sectional and has involved individuals with advanced dementia. However, it can be assumed that if the presence of primitive reflexes is a direct consequence of structural changes in the aging brain, it may be an early sign or accompanying phenomenon of changes in cognitive functioning in older adults. This assumption is supported by empirical and neurobiological evidence linking the integrity of frontal–subcortical circuits with both motor inhibition and executive control. Primitive reflexes are normally suppressed by mature corticospinal and corticobulbar pathways originating from the frontal cortex. Structural and functional deterioration in these regions—such as cortical thinning, white matter degeneration, or reduced inhibitory control—has been consistently associated with both cognitive decline and the re-emergence of reflexive motor patterns in older adults (Jolles et al., 1993; Schott and Rossor, 2003; Vreeling et al., 1995). Therefore, in cognitively intact older adults, the reappearance of primitive reflexes may reflect early behavioral manifestations of subtle cortical disinhibition, which can precede measurable cognitive deficits. This interpretation aligns with longitudinal findings indicating that early disruptions in frontostriatal connectivity predict later cognitive impairment (Miller and Cohen, 2001). Altunkalem Seydi et al. (2024) identified the grasp reflex as the factor with the highest risk of developing dementia in a published study. This meta-analysis showed that the prevalence and risk of primitive reflexes are high in older patients with dementia. Therefore, primitive reflexes, especially the grasp reflex, should be carefully assessed during routine physical examinations as part of the dementia diagnostic process.

Primitive reflexes (PRs) are automatic, brainstem-mediated motor responses that are normally inhibited during early neurodevelopment as cortical control matures (Fiorentino, 2014). Their reappearance or persistence in later life indicates a decline in central inhibitory control and may reflect age-related cortical or subcortical dysfunction (Sanders and Gillig, 2011). Therefore, PRs can serve as accessible behavioral biomarkers of neurocognitive integrity. Assessment of PRs in cognitively healthy older adults allows for the identification of subtle neurobehavioral changes that may precede measurable cognitive decline.

In this context, primitive reflexes could serve as early, non-invasive behavioral indicators of functional brain aging, especially when paired with cognitive assessments such as the MMSE, which reflect frontal lobe-related functions such as attention, memory and orientation.

Based on previous studies, a consistent positive association is demonstrated between physical activity and cognitive functioning. Not only was better cognitive functioning found among more physically active people, but also there is less cognitive decline with age in the active population (Cunningham et al., 2020; Kekäläinen et al., 2023). In addition, physical activity can be associated with fewer persistent primitive reflexes, and it has a protective role against cognitive decline via its effects on primitive reflexes (Stephens-Sarlós et al., 2024, 2025).

In the present study, resilience refers to psychological resilience—an individual’s ability to adapt successfully to stress, adversity, and age-related challenges. Resilience has been identified as a key protective factor for maintaining cognitive health and functional independence in older adults (Windle, 2011; Gooding et al., 2012), and a higher level of psychological resilience is protective against cognitive impairment (Jiang et al., 2024). Therefore, we also included resilience as a potential psychosocial variable to examine whether it relates to primitive reflex activity and cognitive functioning in late life. The association between primitive reflexes and cognitive decline is well-known and well-established, but the pathophysiological and neurobiological basis is not yet fully understood. It can be assumed that further physical and mental factors have an effect on this relationship. The well-documented association between primitive reflexes (PRs) and cognitive decline is supported by clinical and neurobiological studies showing that re-emerging PRs reflect dysfunction within frontal–subcortical inhibitory circuits, which are also affected during aging and dementia. For instance, Vreeling et al. (1995) and Franssen et al. (1993) demonstrated that the frequency of grasp, snout, and palmomental reflexes increases in Alzheimer’s and vascular dementia, correlating with the severity of cognitive impairment. Jolles et al. (1993) similarly observed associations between reflex activity and cognitive aging in non-demented older adults. The pathophysiological basis refers to degenerative or ischemic alterations in cortical and subcortical structures—particularly in the prefrontal cortex, basal ganglia, and their connecting white matter tracts—that reduce inhibitory control over brainstem motor circuits. The neurobiological basis includes mechanisms such as reduced synaptic plasticity, cholinergic dysfunction, and impaired neurotransmission resulting from decreased cerebral blood flow and reduced neurotrophic support (Cotman et al., 2007; Sleiman et al., 2016). Because these neurobiological systems are influenced by modifiable factors such as physical activity, vascular health, stress, and psychological resilience, it is plausible that both physical and mental variables modulate the relationship between primitive reflex expression and cognitive decline. By examining both physical activity and psychological resilience as potential mediators or moderators of the relationship between primitive reflexes and cognitive functioning, this study aims to identify early behavioral markers of brain aging and clarify the modifiable factors that may influence this link. Thus, the present study aimed to examine the moderating/mediating effects of physical activity and resilience on the association between primitive reflexes and cognitive functioning.

## 2 Methods

### 2.1 Study design

This research was designed as a cross-sectional observational study and followed the STROBE (Strengthening the Reporting of Observational Studies in Epidemiology) guidelines (von Elm et al., 2008). The completed STROBE checklist is provided as a Supplementary material.

### 2.2 Study sample and sample size calculation

Participants were recruited from nine residential care facilities for older adults, following approval from the facilities. Eligibility criteria included being at least 60 years of age and physically able to stand and walk without assistance. Exclusion criteria included both physical conditions (e.g., untreated hypertension, cardiovascular problems, dizziness, balance disorders) and psychiatric conditions (e.g., previously diagnosed mental or behavioral disorders). All participants underwent a medical examination, provided informed consent, and signed a GDPR-compliant data management agreement. Of the 115 people who enrolled, 30 participants (80% female; mean age = 73.43 ± 6.85 years) were included in the present study because they completed all assessments and provided fully valid datasets.

The study was conducted in the 2024 Spring/Summer. The study was granted ethical approval by the Regional, Institutional Scientific and Research Ethics Committee, Széchenyi István University (No. DHK-2024/00039/2). The study fully adheres to the ethical principles of the Declaration of Helsinki, and all methods were carried out in accordance with relevant guidelines and regulations.

*A priori* sample size calculation was conducted using G*Power 3.1 (Faul et al., 2007, 2009) for moderation and mediation analyses. The required sample size was computed with t tests, linear multiple regression, fixed model, single regression coefficients and F tests, linear multiple regression, fixed model, *R*^2^ increase. During the estimation (two-tails), the following parameters were set: effect size *f*^2^ = 0.35 (large), alpha error probability = 0.05, Power = 0.95 (Power = 0.85/0.80), number of tested predictors (2, GPAQ and resilience) with two covariates/confounders, BMI and age, the total number of predictors = 4. Based on the results of t-tests and F tests, the required sample size is equal to 40 and 48 (28 and 35; 25 and 31), respectively. Due to the challenge of recruiting independently functioning older people over 65 years, the necessary sample size cannot be achieved only at a lower level of power, which is a limitation of the present study. The *post hoc* computed achieved power was equal to 0.80 and 0.88.

### 2.3 Measurements

#### 2.3.1 Primitive reflexes

Primitive reflexes were assessed using standardized neurological protocols (Fiorentino, 2014; Sanders and Gillig, 2011). A total of 13 reflex responses were tested bilaterally, including Asymmetrical Tonic Neck Reflex (ATNR), Symmetrical Tonic Neck (STNR), Moro, Spinal Galant, Palmar Grasp, Babinski, Core Tendon Guard, Sucking, Tonic Labyrinthine, Babkin response, Glabellar, Post-Rotational Nystagmus (PRN), and Schilder test. Each reflex was assessed in six trials, and a reflex was classified as “present” if it was elicited in at least four trials. The scoring was confirmed by a second observer. As the primary analyses focused on the total number of primitive reflexes, individual reflexes did not demonstrate distinct statistical effects, but their distribution was consistent with previous findings in older populations (Stephens-Sarlós et al., 2025).

#### 2.3.2 Cognitive functioning

Cognitive performance was measured using the Mini-Mental State Examination (MMSE) (Folstein et al., 2001), a widely used screening tool that assesses several areas of cognitive ability, including orientation, immediate and delayed memory, attention, language, and visual-constructive abilities. The MMSE consists of 30 questions, with a maximum score of 30, where lower scores indicate greater cognitive impairment. Despite certain limitations in diagnostic sensitivity, the MMSE remains a reliable tool for cognitive screening in older adults, particularly in community or care settings. All assessments were conducted by PhD students in Health Promotion who were trained in structured procedures for primitive reflex and cognitive functioning assessment under the supervision of a senior researcher, and scores were checked by a second observer to increase reliability.

#### 2.3.3 Physical activity

The level of physical activity was measured with the Global Physical Activity Questionnaire (GPAQ) (World Health Organization, 2002). The GPAQ includes 16 items measuring the frequency, duration and intensity of physical activities in three settings (activity at work, travel to and from places, recreational activities), as well as sedentary behavior. The total physical activity in MET-minutes per week was calculated.

#### 2.3.4 Resilience

Resilience was measured with the Hungarian version of the 10-item Connor–Davidson Resilience Scale (CD-RISC 10) (Connor and Davidson, 2003; Campbell-Sills et al., 2009; Járai et al., 2015). The scale assesses the ability to cope with stress and adversity positively; the total score ranges from 0 to 40, with a higher score indicating a higher level of resilience.

### 2.4 Statistical analysis

**Figure 1:**
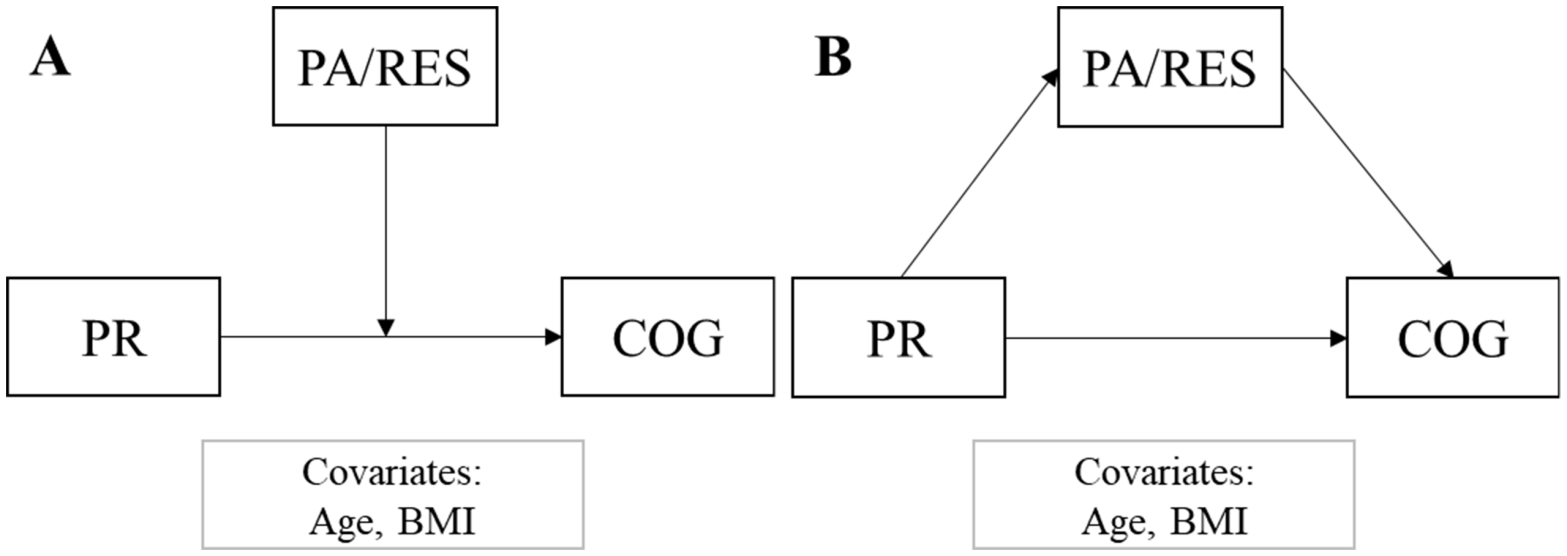
The tested moderation **(A)** and mediation **(B)** of physical activity/resilience on the association between primitive reflexes and cognitive functioning, adjusted for age and BMI. PA, physical activity; RES, resilience; PR, primitive reflexes; COG, cognitive functioning; BMI, body mass index.

For descriptive statistics of the sample size, mean ± standard deviation and relative frequencies were reported. The association between the variables (primitive reflexes, PR; cognitive functioning, COG; physical activity, PA; resilience, RES) was examined using Pearson’s correlation. We tested whether the association between PR and COG is moderated or mediated by PA/RES adjusted for age and BMI (covariates/confounders, Figure 1). Moderation and mediation analyses were conducted with the Hayes’ PROCESS macro (Hayes, 2022). For simple moderation, Model 1, for simple mediation, Model 4 was used with the covariates/confounders. The level of significance was set at 0.05.

## 3 Results

### 3.1 Study sample characteristics

The study sample included 30 older adults (80% female) aged between 62 and 89 (M = 73.43 ± 6.85). Table 1 includes the study sample characteristics of the measured variables.

**Table 1:** Study sample characteristics on the measured variables (*n* = 30).

Characteristics	Frequency/M ± SD
Female sex, % (*N*)	80.00 (24)
Age, M ± SD	73.43 ± 6.85
BMI, M ± SD	26.23 ± 3.23
PRnr, M ± SD	9.00 ± 3.53
MMSE, M ± SD	23.57 ± 3.51
GPAQ, M ± SD	1374.93 ± 758.992
RES, M ± SD	29.97 ± 5.35

BMI, body mass index; PRnr, the number of primitive reflexes; MMSE, Mini-Mental State Examination; GPAQ, Global Physical Activity Questionnaire; RES, 10-item Connor–Davidson Resilience Scale.

Among these, the most frequently observed reflexes in our sample were the Schilder-test, Tonic Labyrinthine, and Babkin reflexes (Table 2).

**Table 2:** The prevalence of the 13 primitive reflexes.

Primitive reflexes	Right		Left	
	*n*	%	*n*	%
Schilder-test	21	70.0	21	70.0
Tonic Labyrinthine	19	63.3	19	63.3
Babkin response	15	50.0	12	40.0
Asymmetrical Tonic Neck Reflex	13	43.3	17	56.7
Core Tendon Guard	13	43.3	12	40.0
Moro	11	36.7	8	26.7
Babinski	9	30.0	7	23.3
Glabellar	8	26.7	8	26.7
Post-Rotational Nystagmus	8	26.7	8	26.7
Palmar Grasp	7	23.3	4	13.3
Symmetrical Tonic Neck	6	20.0	6	20.0
Sucking	6	20.0	2	6.7
Spinal Galant	5	16.7	5	16.7

### 3.2 Association between primitive reflexes, cognitive functioning, physical activity, and resilience

The number of primitive reflexes and cognitive functioning revealed a statistically significant, negative very strong association [*r*(28) = −0.904, *p* < 0.001]. The number of primitive reflexes also showed a significant, negative strong association with resilience [*r*(28) = −0.763, *p* < 0.001] and a negative moderate association with physical activity [*r*(28) = −0.598, *p* < 0.001]. In addition, cognitive functioning is significantly associated (positive strong) with physical activity [*r*(28) = 0.709, *p* < 0.001] and resilience [*r*(28) = 0.782, *p* < 0.001]. Finally, physical activity and resilience also showed a significant, positive strong correlation [*r*(28) = 0.794, *p* < 0.001].

### 3.3 The role of physical activity and resilience in the association between primitive reflexes and cognitive functioning

#### 3.3.1 Moderation models

**Figure 2:**
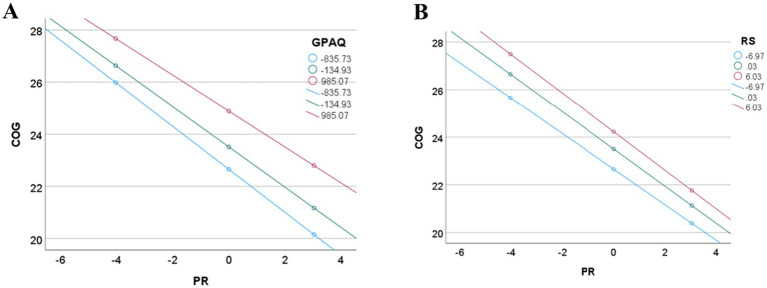
The moderating effect of physical activity **(A)** and resilience **(B)** in the association between primitive reflexes and cognitive functioning.

Physical activity and primitive reflexes were significant independent predictors of cognitive functioning, with 90% of explained variance [*F*(5,24) = 43.368, *p* < 0.001]. At the same time, the interaction, the moderation effect was not significant (PR*PA, t = 0.680, *p* = 0.503). In this model, the covariates, BMI (*t* = −2.415, *p* = 0.024) and age (*t* = 2.180, *p* = 0.039), were also significant (Figure 2A).

The model, testing the moderating effect of resilience, was significant [*F*(5,24) = 32.569, *p* < 0.001]; however, only the number of primitive reflexes was a significant independent predictor (*t* = −6.452, *p* < 0.001), and BMI as a covariate (*t* = −2.351, *p* = 0.027) (Figure 2B).

#### 3.3.2 Mediation models

The model, using resilience as a mediator, in sum, was significant [*F*(4,25) = 42.108, *p* < 0.001]. However, only the number of primitive reflexes was a significant predictor of cognitive functioning (*t* = −6.558, *p* < 0.001). Resilience did not show a mediating, indirect effect (*β* = −0.143, bootstrapped 95% CI = −0.412; 0.024).

**Figure 3:**
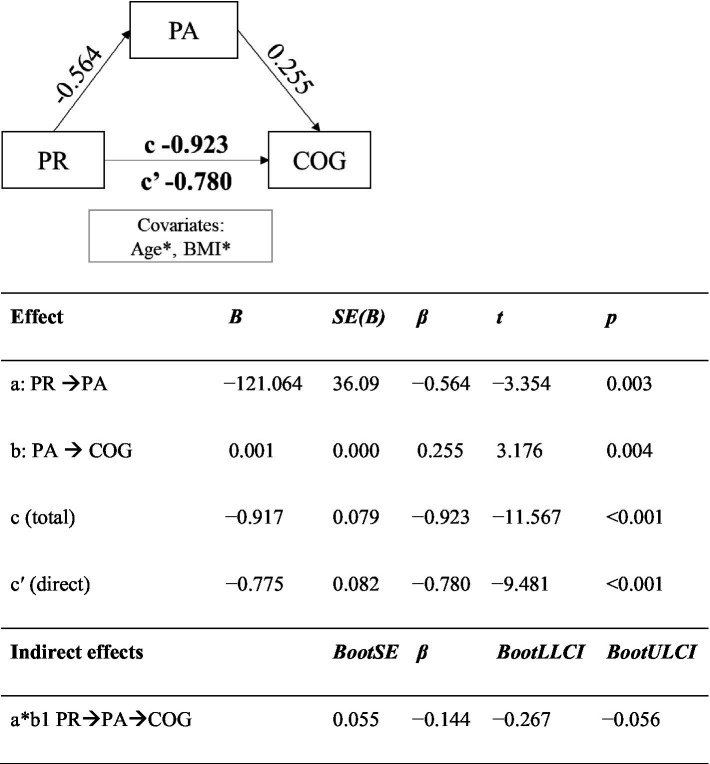
The mediating role of physical activity in the association between primitive reflexes and cognitive functioning, adjusted for age and BMI. PR, the number of primitive reflexes; COG, cognitive functioning; PA, physical activity; *B*, coefficient; *β*, standardized; SE, standard error; BootLLCI, 95% bootstrapped confidence interval lower level; BootULCI, 95% bootstrapped confidence interval upper level; ^*^significant covariates in mediation model: Age, 0.09 (0.01; 0.16); BMI, −0.19 (−0.34; −0.03).

The results revealed a significant indirect effect of PR on COG through PA. At the same time, the direct effect of PR on COG in the presence of a mediator, using age and BMI as covariates, was also significant. PA partially mediated the association between PR and COG. Mediation is presented in Figure 3 with the summary table of the effects.

## 4 Discussion

This study investigated the associations between primitive reflexes, cognitive functioning, physical activity, and resilience in older adults. The results confirmed that a higher number of primitive reflexes and lower physical activity independently indicate lower cognitive functioning, taking into account BMI and age. Physical activity may mediate the association between primitive reflexes and cognitive functioning. Although resilience was associated with primitive reflexes and cognitive functioning, it did not have a moderating or mediating effect. These findings suggest that while both physical and psychological factors relate to cognitive health in aging, only physical activity exerts a statistically significant pathway through which primitive reflexes may influence cognition.

Based on previous studies, resilience is a protective factor for healthy aging (Gooding et al., 2012). This protective role is thought to operate through several biopsychosocial pathways, including lower levels of chronic inflammation and oxidative stress (Majnarić et al., 2021), fewer symptoms of depression and anxiety with better emotional regulation and neural plasticity (Ong et al., 2006; Gooding et al., 2012; Feder et al., 2009; Russo et al., 2012), and greater engagement in health-promoting behaviors such as physical activity and social participation (Windle, 2011; Netuveli et al., 2008). Although resilience did not show a significant mediating or moderating effect in our models, its strong correlations with both cognitivThisprtee performance and physical activity suggest an indirect, supportive role within broader psychosocial aging processes. Higher psychological resilience is associated with a lower risk of age-related chronic diseases and mortality, as well as a slower decline in physical and mental health and longer life expectancy (Gooding et al., 2012; Hayman et al., 2017; Majnarić et al., 2021). Physical activity is positively associated with resilience, and previous cross-sectional studies suggest that this association might depend on exercise volume. In the present study, physical activity was assessed using the Global Physical Activity Questionnaire (GPAQ; World Health Organization, 2002), which captures total weekly activity across work, transport, and recreational domains, expressed in MET-minutes per week. Therefore, exercise volume refers to the total amount of physical activity (duration × intensity × frequency) rather than a specific exercise type. Most participants engaged in low- to moderate-intensity activities, such as walking or light group exercises, commonly practiced in residential care settings. Besides the additional positive physical and mental effects of physical activity, maintaining an active lifestyle in older age can also result in a higher level of resilience (Toth et al., 2023). Although resilience did not emerge as a significant moderator or mediator in this study, its strong associations with both cognitive and motor indicators suggest that it may play an indirect role within broader psychosocial aging processes.

Research findings show that the frequency and expression of primitive reflexes increase with age, especially after the age of 70, and in conjunction with cognitive decline. Recent neurogerontological studies have confirmed that the reappearance of nociceptive primitive reflexes (such as glabellar, snout, and palmomental reflexes) is frequent even in neurologically and cognitively healthy aging individuals and correlates with subtle declines in executive and attentional functions (van Boxtel et al., 2006; Camarda et al., 2019). This reactivation is thought to reflect frontal disinhibition and white matter degeneration, as age-related structural changes—such as white matter hyperintensities, caudate atrophy, and cortical thinning—disrupt the inhibitory control of subcortical circuits (Camarda et al., 2019).

Parallel to the decline in cognitive abilities, there are significant changes in the mass and structure of striated muscle due to physiological aging, or the development of lesions associated with more severe (degenerative) joint inflammation (Rabbitt et al., 2001; Núñez-Lisboa et al., 2023). According to recent biomechanical and neurophysiological reviews, neurodegenerative changes within motor pathways—including loss of spinal motor neurons, reduced neuromuscular junction efficiency, and altered descending control—play a key role in the functional decline of motor performance with age (Núñez-Lisboa et al., 2023).

A strong association was found between primitive reflexes and resilience, indicating that lower adaptive capacity is linked to reduced motor control and stability in older adults. Resilience was also positively correlated with physical activity and cognitive functioning, highlighting its relevance in maintaining balance and overall functional performance in aging. The significance of age-related changes in muscle function (Cruz-Jentoft and Sayer, 2019) is dramatically increasing due to socio-economic factors related to demographic developments in modern society, which are leading to an increase in the proportion of older people as a result of improved living conditions and advances in healthcare (Lord et al., 2021; Gustafsson et al., 2024). Another important factor related to falls and fall-related injuries in older adults should not be overlooked. Its morphological background is closely related to the above-mentioned factors, but it is also much more complex. Falls can be divided into two components: the onset of the fall and the ability to restore balance (Xing et al., 2023). Age-related changes in somatosensory perception, vision, and vestibular function have a negative impact on maintaining postural balance, which increases the risk of falls. These effects are significant on their own, but when combined, they place a heavy burden on care systems (Anton et al., 2015).

### 4.1 Implications and future directions

Physical activity has numerous beneficial effects on the brain: (1) it improves cognitive function, (2) it improves blood circulation, and (3) it reduces the number of falls and the severity of injuries resulting from them. One mechanism explaining changes in brain plasticity is the effect of growth factors (Cotman et al., 2007). Signaling pathways of brain-derived neurotrophic factors (BDNF) play an important role in learning and memory formation. Although BDNF was not directly measured in this study, it is discussed as a theoretical mechanism supported by prior evidence linking physical activity and neuroplasticity. Previous studies have demonstrated that regular exercise and other lifestyle factors can enhance BDNF signaling, contributing to cognitive resilience and healthy brain aging (Colcombe and Kramer, 2003; Marosi and Mattson, 2014). It has been known for more than two decades that physical activity or nerve cell activity significantly increases BDNF gene expression in the brain (Isackson et al., 1991; Neeper et al., 1995) and that an increase in BDNF protein leads to the activation of signaling pathways that are associated with exercise-induced improvements in learning and memory formation (Vaynman et al., 2004; Sleiman et al., 2016). These neurotrophic and vascular mechanisms may help preserve cortical inhibition over brainstem circuits, potentially reducing the reactivation of primitive reflexes in later life. Although these findings are widely recognized, it is important to note that very little is known about the molecular mechanisms linking exercise and BDNF expression. Recent reviews (Di Liegro et al., 2019) emphasize that, despite the strong association between exercise and BDNF upregulation, the specific molecular pathways remain under-elucidated, highlighting the need for further studies to clarify how exercise-induced molecular cascades contribute to neuroplasticity and functional preservation in aging.

### 4.2 Limitations

The frequency of primitive reflexes varies greatly, and there is no consensus on their pathological significance. Furthermore, there are differing opinions as to whether their occurrence becomes more frequent with aging. Only the grasp reflex and the plantar extensor reaction (Babinski sign) have been proven to be indicators of central nervous system diseases. These controversies may be explained by conceptual and methodological differences between authors. For example, with regard to the interpretation of motor reactions, some consider only lip retraction to be a positive sucking reflex, while others require additional tongue and larynx sucking movements.

There may also be differences in the variables influencing the occurrence of primitive reflexes, such as the heterogeneity of diseases occurring in the patient groups studied, the lack of standardized, quantified protocols, the intensity of stimulation, and the emotional state of the person being tested, all of which may influence the magnitude and duration of responses. It is important to note that the sample size of our study is relatively small. Additional covariates and confounding factors include social support, comorbidity, chronic diseases, and medication use.

The relatively small sample size and the predominance of female participants limit the generalizability of the findings; therefore, the results should be interpreted as preliminary and indicative. Furthermore, potential effects of postmenopausal hormonal changes on cognitive and neural function were not assessed and represent an important direction for future research.

## 5 Conclusion

The present study aimed to examine the moderating/mediating effects of physical activity and resilience on the association between primitive reflexes and cognitive functioning. The findings revealed that the higher number of primitive reflexes and lower physical activity indicate lower levels of cognitive functioning. It also showed that physical activity may mediate the association between primitive reflexes and cognitive functioning. Even though resilience was associated with primitive reflexes and cognitive functioning did not have a moderating or mediating effect. These findings highlight the importance of physical activity not only as a cognitive protective factor but also as a possible modulator of brain–body interactions such as primitive reflex regulation. Integrating reflex assessment into routine geriatric evaluation may offer a novel behavioral tool to detect early functional decline. As there are currently no specific programs to improve resilience in older adults, we support the creation of an integrated healthcare infrastructure that would provide comprehensive care programs for the elderly population. In addition, promoting physical activity may help mitigate neurocognitive decline, supporting integrative strategies for healthy brain aging.

## Data Availability

The raw data supporting the conclusions of this article will be made available by the authors, without undue reservation.
